# Moving on obesity treatment in CKD: inertia is unjustified

**DOI:** 10.1093/ckj/sfad275

**Published:** 2023-11-22

**Authors:** Carmine Zoccali

**Affiliations:** Renal Research Institute NY, USA; BIOGEM, Ariano Irpino, Italy; IPNET, Reggio Cal, Italy

The ketogenic diet has its origins in the early 20th century as a treatment for drug-resistant epilepsy. Developed initially in the 1920s, the diet aimed to mimic the biochemical effects of fasting, which had been observed to reduce seizure frequency [1]. By restricting carbohydrate intake and increasing fat consumption, the diet induces a metabolic state known as ketosis. In ketosis, the body relies on the conversion of fatty acids into ketones for energy, which can be utilized by the brain and other tissues. Over the years, the application of the ketogenic diet has extended beyond epilepsy to include a variety of medical conditions and lifestyle goals, most notably obesity and diabetes [2]. This diet showed benefits in experimental models of autosomal dominant polycystic kidney disease (ADPKD) [3] and has been proposed as a possible treatment for this disease in humans [4].

Because of the very high proportion of overweight and obese patients in the chronic kidney disease (CKD) population [5], the application of a ketogenic diet in obese patients with stage 1–3a CKD, which is a large fraction (at least 40%) of the CKD population [6, 7], may be an interesting option. Indeed, the rapid weight loss effects of the ketogenic diet make it an attractive intervention for this patient population. Proponents suggest that in addition to its efficacy in weight loss, there are added metabolic advantages, including improved insulin sensitivity and a decrease in systemic inflammation [8].

However, reservations exist about the suitability of the ketogenic diet in CKD 1–3a patients [9]. Critics argue that the diet's high protein and fat content could pose additional metabolic stress on already compromised kidneys. Furthermore, the diet restricts the intake of carbohydrates, including fruits and vegetables, that provide essential nutrients, potentially leading to nutritional deficiencies.

Given these conflicting viewpoints, the issue of whether the ketogenic diet is an appropriate, safe and effective intervention for weight management in stage 1–3a CKD patients remains a topic of ongoing scientific debate [10].

In this *CKJ* controversy, Joshi *et al.* [11] discuss the potential risks of the ketogenic diet in CKD. These authors stress that metabolic acidosis, a common consequence of a ketogenic diet [12], increases the risk for a range of complications, including bone disease and muscle wasting in CKD [13]. Additionally, the ketogenic diet restricts carbohydrate-dense plant foods that are associated with reduced cardiovascular disease mortality and all-cause mortality. Overall, Joshi *et al.* [11] suggest that the ketogenic diet may not be a safe or effective dietary option for individuals with CKD. In their view, in individuals with CKD, nephrologists should focus on a balanced diet that includes a variety of nutrient-dense foods, including fruits, vegetables, whole grains and lean protein sources. Dietary counselling and support may also be helpful for promoting adherence to a healthy diet such as the Mediterranean or plant-dominant low protein diet (PLADO) diets [14]. Plant foods have several potential benefits for patients with CKD, and the alkali found in these foods may help prevent worsening of metabolic acidosis or the development of nephrolithiasis. Examples of plant fats for consideration by patients with CKD on a ketogenic diet include avocados, oils (i.e. olive oil and canola oil), nuts and seeds.

The opponents, Weimbs *et al*. [15], support the prescription of ketogenic metabolic therapy (KMT) in CKD patients, a dietary approach that involves consuming a high-fat, low-carbohydrate diet to induce a state of ketosis in the body. They state that KMT may help to improve glycaemic control, reduce inflammation and promote weight loss.

KMT may not only decrease baseline blood glucose levels, but may also reduce glycaemic spikes, which can help to prevent further kidney function loss in CKD patients. They stress that numerous studies suggest that ketogenic diets are also more effective for inducing weight loss and decreasing hypertension than low-fat diets [15]. The pros and cons of KMT are summarized in Table 1.

**Table 1:** Pros and cons of a ketogenic diet in CKD patients.

Pros	Cons
**Rapid weight loss: ** The diet can induce rapid weight loss, which is particularly useful for the high proportion of overweight and obese CKD patients [5–7]	**Metabolic stress on kidneys: ** The diet's high protein and fat content could pose additional stress on already compromised kidneys [9]
**Improved insulin sensitivity: ** The diet improves insulin sensitivity and decreases systemic inflammation ,which is beneficial for metabolic health [8]	**Nutritional deficiencies: ** The diet restricts the intake of carbohydrates, including fruits and vegetables, potentially leading to nutritional deficiencies [9]
**Reduced inflammation: ** KMT may help to reduce inflammation [15]	**Metabolic acidosis: ** Metabolic acidosis, a feature of the ketogenic diet, could lead to bone disease and muscle wasting in CKD patients [12, 13]
**Glycaemic control: ** KMT improves glycaemic control and may prevent further kidney function loss [15]	**Reduced cardiovascular health: ** The diet restricts carbohydrate-dense plant foods associated with reduced cardiovascular disease mortality and all-cause mortality [11]
**Effective in hypertension: ** Ketogenic diets are more effective for reducing hypertension than low-fat diets [15]	**Risk of kidney stones: ** Concerns exist about the potential risks, such as nutrient deficiencies and increased risk of kidney stones
**High adherence rate: ** Data show that these diets may warrant a high adherence in the long term [18]	**Insufficient long-term data: ** There is limited research on the long-term effects of KMT on CKD patients. Clinical trials are needed to test the safety and effectiveness [16, 17]

This moderator remarks that there is still limited information on the long-term effects of KMT on CKD patients. Some highly reputed experts have raised concerns about the potential risks of the diet, such as nutrient deficiencies and an increased risk of kidney stones [10]. Full-fledged, well-powered clinical trials are still needed to thoroughly test the safety and effectiveness of KMT for weight loss in CKD patients. Importantly, these trials should also test the effect of weight loss on long-term kidney function because the early and persistent (up 2 years) increase in the estimated glomerular filtration rate (eGFR) observed in a trial by Israeli investigators [16] may be the result of hyperfiltration secondary to increased protein intake. However, in this trial KMT reduced albuminuria (hyperfiltration should normally increase it), which makes it unlikely that this regimen may damage the kidney.

In brief, for half a century renal nutrition has centred on limiting protein intake as renal function declines in CKD. Current guidelines recommend limiting protein intake to 0.8–1.0 g/day only when the eGFR is <30 ml/min/1.73 m^2^ [17]. Since obesity is a dominant problem from stage G1 CKD onwards [6, 7] and a risk factor for CKD progression [18], this moderator believes that the time is ripe for experts on renal nutrition to test dietary treatments with low or very low carbohydrate content and high fat and a moderately increased protein content in obese CKD patients. Data gathered so far show that this approach warrants high adherence in the long term [16]. Obesity is probably the most concerning risk factor for human health [6] and an inertial attitude by nephrologists on this problem is unjustified.
