# Correction to: Moving on obesity treatment in CKD: inertia is unjustified

**DOI:** 10.1093/ckj/sfae149

**Published:** 2024-05-24

This is a correction to: Carmine Zoccali, Moving on obesity treatment in CKD: inertia is unjustified, *Clinical Kidney Journal*, Volume 17, Issue 1, January 2024, sfad275, https://doi.org/10.1093/ckj/sfad275

In the originally published version of this article, a typographical error was present in the in-text citations of ‘Joshi S, Shi R, Patel J. Risk of ketogenic diet in CKD. *Clin Kidney J* 2023;**17**:1–5. https://doi.org/10.1093/ckj/sfad274’.

This has been corrected.

